# Tattoo Pigment In Situ as a Determinant of Laser Treatment Response: Composition, Particle Size Distribution, Cellular Localisation, and In Situ Transformations

**DOI:** 10.3390/jcm15197593

**Published:** 2026-09-30

**Authors:** Sylwia Dobrowolska, Paulina Wojtyła-Buciora, Maria Czwojda, Sylwia Nawrot, Tomasz Hadada, Edyta Mądry, Joanna Bartkowiak-Wieczorek

**Affiliations:** 1 Amazink Sp. z o.o., ul. Wojskowa 6/D11, 60-792 Poznan, Poland; amazinkareola@gmail.com (S.D.); drsylwianawrot@gmail.com (S.N.); 2 Faculty of Medicine and Health Sciences, Calisia University, 62-800 Kalisz, Poland; p.wojtyla-buciora@uniwersytetkaliski.edu.pl; 3 Department of Physiology, Poznań University of Medical Sciences, 61-701 Poznan, Poland; thadada@ump.edu.pl (T.H.); emadry@ump.edu.pl (E.M.); 4 Department of Adult Psychiatry, Poznań University of Medical Sciences, 60-572 Poznan, Poland; mczwojda@skp.ump.edu.pl; 5 MLODOSC Clinic, Libelta 27/18A, 61-707 Poznan, Poland

**Keywords:** tattoo, permanent makeup, pigment, nanoparticles, aromatic amines, REACH, titanium dioxide, carbon black

## Abstract

**Introduction:** The safety assessment of tattoo removal procedures rests on the assumption that the treatment substrate corresponds to the ink composition declared by the manufacturer. This assumption has rarely been verified. **Methods:** A structured, comprehensive literature search was conducted across PubMed/MEDLINE, Embase, Scopus, and Web of Science using a four-block strategy encompassing ink composition and impurities, particle characteristics, cellular localisation, and in situ transformations. Analytical chemistry studies, tissue imaging, market composition research, and regulatory documentation were included. **Results:** The material in the skin may differ from product documentation in four respects. A submicron fraction exists in commercial inks before any procedure. Many products contain undeclared ingredients, and aromatic amines may occur as manufacturing impurities before irradiation. The deposit is probably not inert: photodegradation has been shown outside tissue and remodelling in mice, whereas oxidation remains hypothetical. Finally, Colour Index numbers and INCI declarations do not unambiguously identify the substance. **Conclusions:** The substrate of the removal procedure remains incompletely characterised and may, depending on the age of the tattoo, predate the harmonisation of ink composition in the European Union. The evidence documents chemical hazards rather than demonstrated clinical risk, with consequences for literature inclusion criteria, the design of degradation studies and informed consent.

## 1. Introduction

Tattooing has ceased to be a marginal, subcultural practice and has become a common form of permanent body modification among populations in highly developed countries [1]. A review covering 86 studies reports a prevalence ranging from 5.2% to 35.3% in developed countries, with a statistically significant upward trend and a marked over-representation of younger age groups [2]. Behind these figures lies a growing population permanently exposed to mixtures of industrially produced colourants deposited in the dermis and, proportionally, a growing demand for removal procedures [2].

Both the safety assessment of tattooing itself and that of its removal rest on a shared assumption that is rarely stated explicitly: that the material present in the skin corresponds to what the ink manufacturer has declared, an assumption implicit, for example, in experimental studies of laser-induced decomposition that start from identified pigments [3,4]. Annex XVII to the REACH Regulation (EC) No 1907/2006, however, sets out restrictions on the manufacture, placing on the market and use of hazardous substances, mixtures and articles, and its entry 75, added by Commission Regulation (EU) 2020/2081 [5], regulates the composition of tattoo and permanent make-up inks. Entry 75 covers more than 4000 substances, with thresholds of 0.00005% for carcinogens and mutagens, 0.001% for reproductive toxicants and skin sensitisers, and 0.01% for corrosive and irritant substances, and it also imposes a labelling requirement. The limits were based on the hazard classes of the substance placed on the market rather than on its degradation products [6]. The effectiveness of these provisions nevertheless remains limited: among 41 inks purchased on the EU market after the restriction entered into force, the limits were exceeded for nickel in 24 samples, arsenic in 20 and chromium (VI), estimated as 1% of total chromium, in 16, and the modelled lifetime cancer risk exceeded 1 × 10^−4^ for nickel in several products [7].

There are, therefore, grounds to conclude that the contents of a pigment or ink bottle do not correspond to the label. Of 54 inks available on the US market, 45 contained undeclared pigments or additives, including polyethene glycol, propylene glycol and higher alkanes [8], and of ten inks declared to be REACH-compliant, nine proved non-compliant, four of them containing banned material, while the polymorph of Pigment Blue 15 could not be unambiguously determined [9]. Market surveillance yields a convergent picture: in a European enforcement action covering 52 inks, 27 samples showed a non-compliant composition, and 10 showed non-compliant labelling [10].

Irrespective of the quality of labelling, the material deposited in the skin ceases to be identical with the starting product at the very moment of injection. As shown in post-mortem human tissue, it undergoes size fractionation and drainage to the regional lymph nodes, with organic pigments displaying the widest size range and the smallest particles migrating preferentially; in parallel, ultrastructural changes occur in the tissue adjacent to the pigment particles, expressed as an altered α-helix/β-sheet conformational ratio in the amide I band [11]. The pigment is simultaneously taken up by dermal macrophages and, as shown in a mouse model, retained there through cycles of uptake, release, and re-uptake that outlast the lifespan of a single cell, although the applicability of this mechanism to human skin has not been determined [12]. A suspension of fresh pigment and a deposit present in the skin after years differ in composition, particle size distribution and cellular context; that is, in parameters that, on physical grounds, can be expected to determine the response to a laser pulse [11].

The aim of this review is to establish what is present in the skin of a tattooed person before any tattoo removal intervention, and to assess the extent to which this material remains identifiable. The work comprises a characterisation of the chemical classes of pigments together with the particle size distribution and aggregation state; a determination of whether a submicron fraction exists in the deposit independently of laser intervention; and an assessment of the presence of non-pigment constituents, metallic impurities, polycyclic aromatic hydrocarbons and aromatic amines prior to irradiation, thereby establishing the baseline against which the effects of the procedure can be judged. Throughout the review, chemical hazard, understood as the intrinsic capacity of a substance to cause harm, is distinguished from risk, which also depends on the dose actually received, and from clinical risk, which requires adverse outcomes to be demonstrated in exposed people; the review addresses the identity of the material and the hazards associated with it, not the quantification of clinical risk.

## 2. Literature Search Strategy and Synthesis

This work is a comprehensive narrative and critical review. It does not claim compliance with the PRISMA 2020 statement [13], which was developed for systematic reviews of clinical intervention studies and is not applicable to a cross-disciplinary synthesis of analytical chemistry, imaging, histological and regulatory evidence. The search strategy is nevertheless reported in full below, and the search strings for each database are given in Appendix A (Table A1), so that it can be reproduced independently, although the reported yields can be verified only for the PubMed search.

The protocol was developed jointly by all authors. Database searching was carried out by S.D. and J.B.-W. Screening and data extraction were performed independently by three reviewers, S.D., T.H. and S.N.; disagreements not resolved through discussion were adjudicated by J.B.-W. acting as arbiter. The inclusion criteria and the extraction form were calibrated in collaboration with P.W.-B., J.B.-W., and E.M.

Four bibliographic databases were searched: PubMed/MEDLINE via the NCBI interface, Embase via the Elsevier platform, Scopus and the Web of Science Core Collection.

This choice was dictated by the subject matter being dispersed across three bodies of literature with incomplete mutual coverage. The dermatological literature is best indexed in MEDLINE. The analytical chemistry and materials science literature, in which tattooing appears not as a clinical topic but as the material under investigation, is indexed more broadly in Scopus and Web of Science. Embase was included because of its broader coverage, relative to MEDLINE, of toxicological journals, European dermatological titles with national circulation, and conference abstracts, which in this field carry a substantial share of the analytical data. Regulatory documentation was not sought in the bibliographic databases because it is not indexed there; it was covered by a separate targeted search.

The date of the last search in all four databases was 14 August 2026. No lower date limit was applied in the main search. No publication-type or methodological filters were applied, because a substantial part of the analytical-chemical data appears in formats that such filters exclude; selection by study type was performed only at the full-text assessment stage.

Searching was conducted in the title and abstract fields. The strategy was constructed from a population core and four thematic blocks. The core was combined with each block using the AND operator, and the blocks were combined with one another using the OR operator. In total, the strategy comprises thirty-nine search expressions.

The core comprised six expressions: tattoo, tattoos, tattooing, tattooed, permanent make-up and permanent makeup. Both spelling variants of the term denoting permanent make-up had to be included because, in the English-language literature, they are unevenly distributed between European and American journals.

Block A: composition, impurities and labelling

The block comprises ten expressions in two subgroups. The first subgroup specifies the material: ink*, pigment*. The second specifies the object of analysis: composition, ingredient*, impurity*, heavy metal*, aromatic amine*, polycyclic aromatic, label* and labelling. The two subgroups were combined using AND, which distinguishes studies on ink composition from those in which the term pigment refers to melanin.

Block B: physicochemical characterisation of the particle

The block comprises six expressions: nanoparticle*, particle size, nanomaterial*, aggregat*, agglomerat* and surface area. Separating aggregation from agglomeration is deliberate because in the materials science literature, the two terms are not synonymous, and the distinction bears on the interpretation of particle behaviour in tissue.

Block C: tissue and cellular localisation

The block comprises eight expressions: macrophage*, fibroblast*, mast cell*, keratinocyte*, dendritic, intracellular, epidermis and dermis.

The set of cell types corresponds directly to the findings of Kröger et al. [14], who used two-photon excited fluorescence lifetime imaging to demonstrate in vivo the presence of pigment particles in macrophages, mast cells and fibroblasts of the dermis, and in keratinocytes, dendritic cells and basal layer cells of the epidermis, that is, also in cell types rarely considered in this context. The inclusion of mast cells and keratinocytes alongside the cell types routinely associated with the pigment deposit, therefore, followed from evidence available before the strategy was constructed, and not from a modification introduced during searching.

Block C has the lowest specificity of the four, because the expressions *dermis* and *epidermis* capture most histological studies on tattooing, regardless of whether they address pigment localisation.

Block D: transformations occurring in situ

Block D comprises nine expressions: photodegradation, photostability, photodecomposition, sunlight, ultraviolet, UV, metabolism, ageing and ageing. Both spelling variants of the term denoting ageing had to be included due to differences between British and American orthography.

Structure of the retrieved set

The structure of the retrieved set was analysed in PubMed on 14 August 2026.

The population core without thematic restriction returned 6896 records. The combination with the thematic blocks yielded 151 records for block A, 151 for block B, 321 for block C, and 138 for block D. The identical yields for blocks A and B are a coincidence, confirmed by independent repetition of the queries; the blocks share no expressions, and their intersection contains 24 records.

The arithmetic sum of the four blocks is 761 records, whereas the logical union after removal of duplicates is 639 records. The difference of 122 corresponds to the number of repeated record occurrences across blocks, not to the number of records belonging to more than one block: a record present in three blocks contributes two repetitions to this difference while remaining a single record. The number of records meeting the criteria of more than one block is 97, or 15.2% of the deduplicated set. The distribution of block membership is presented in Table 1; it confirms that the blocks describe distinct, although partly overlapping, areas of the literature.

The largest intersection is that between blocks B and C (35 records) and corresponds to imaging studies in which particle size characterisation was performed together with determination of cellular localisation, that is, to the studies closest to the subject of the present review (Table 2).

Restricting the set to the years 2000–2026 reduced it to 544 records, thereby discarding 95 records published before 2000. This restriction was not applied in the main search; it was performed solely as a sensitivity analysis, because some of the foundational studies on pigment localisation in the skin date from the 1960s, 1980s and 1990s and have no later counterparts.

Supplementary search procedures

Procedures supplementary to the database searches were also carried out.

The reference lists of all studies included at the full-text stage were screened. Studies citing ten publications regarded as key to the subject were checked; because the list of these seed publications was not available for this revision, this step cannot be reproduced. Regulatory documentation was searched: Commission Regulation (EU) 2020/2081, the Joint Research Centre report on the safety of inks, European Chemicals Agency documentation and Council of Europe Resolution ResAP(2008)1. Standard EN 17169:2020 and national implementing acts were examined insofar as they relate to product composition.

Eligibility criteria

Studies were included in the review if they met all three of the following conditions.

First, the subject of the study had to be tattoo or permanent make-up ink, tattoo pigment, or the pigment deposit in the skin prior to any removal intervention. Second, the study had to address at least one of five topics:

1. chemical composition and impurities;
2. physicochemical characterisation of the particle, including size distribution, aggregation and specific surface area;
3. tissue and cellular localisation of the pigment;
4. transformations of the deposit occurring in the absence of any removal procedure;
5. agreement between the composition declared by the manufacturer and the actual composition.

Third, the study had to belong to one of the following types: analytical chemistry study, market survey of product composition, in vivo or ex vivo tissue imaging, histological study, animal model study of pigment localisation or retention, systematic review, regulatory document or standard.

A language restriction was applied to studies in English, Polish, German, and French, corresponding to the team’s language competencies. The resulting risk of missing data is addressed in the Discussion.

Publication date as a potential exclusion criterion

A separate decision was required as to whether studies concerning pigments banned or restricted after 4 January 2022, that is, after the entry into force of entry 75 of Annex XVII to the REACH Regulation, should be excluded as outdated.

The change in ink composition imposed by the REACH Regulation does not render earlier publications obsolete, including studies on pigments banned after 2022. This follows from the temporal asymmetry between regulation and exposure: the provision takes effect when the product is placed on the market, whereas exposure begins when the material is introduced into the skin and continues thereafter. Consequently, the set of pigments present in the skin of the European population in 2026 includes those derived from products sold before 4 January 2022, that is, under the pre-harmonisation regime, in which most Member States were governed at most by the non-binding Resolution ResAP(2008)1 [15].

It was therefore assumed that the date of publication does not constitute an exclusion criterion. The justification has three parts.

First, the subject of the review is ink in the skin, not ink in the commercial container. Entry 75 regulates only the placing of products on the market; it is not retroactive and removes nothing from the tissues of people tattooed before it entered into force.

Second, the age structure of the treatment population determines the composition of the substrate, but it cannot be derived from the data cited here. The median of 14 years reported in a British study of 68 patients requesting laser removal, published in 1999, refers to the duration of regret before removal was sought, not to the time from having a tattoo made to presenting for its removal [16]. In an Italian population survey completed in 2015, 36.7% of all tattoos had been made within the 5 years preceding the interview [17], but this figure describes the tattooed population as a whole rather than persons seeking removal. Neither source, therefore, allows the age or the regulatory origin of tattoos removed in 2026 to be determined, and excluding studies on pigments restricted after 2022 would rest on the unverified assumption that such pigments are no longer present in the treatment population.

Third, data from the period after 2022 do not confirm a complete turnover of the pigment pool on the market: non-compliant or banned material was still found both in inks declared to be REACH-compliant [9] and in a European enforcement action [10] (Section 1).

Every study concerning a currently restricted pigment was therefore included with an explicit indication of that pigment’s regulatory status, assigned to one of the three categories defined on the basis of entry 75 of Annex XVII to the REACH Regulation and indicated for each pigment named in the cited studies in Appendix A (Table A3).

Records retrieved and included

The PubMed search of 14 August 2026 returned 639 records, and re-running the PubMed strings on 10 September 2026, with the entry date limited to 14 August 2026, reproduced the yields reported above to within two records, a difference attributable to updates of individual PubMed records. The yields of the searches in Embase, Scopus and the Web of Science Core Collection were not available for this revision and are therefore not reported; the corresponding strings are listed in Appendix A (Table A1) so that these searches can be re-run. The core synthesis comprises 33 publications and documents (Appendix A, Table A2). Of these, 29 are indexed in PubMed, and 23 of them are contained in the re-run PubMed result set; the remaining six [18,19,20,21,22,23] and the four documents not indexed in PubMed [5,10,16,24] were identified through the other databases or the supplementary procedures.

Appraisal of the evidence

**Figure 1:**
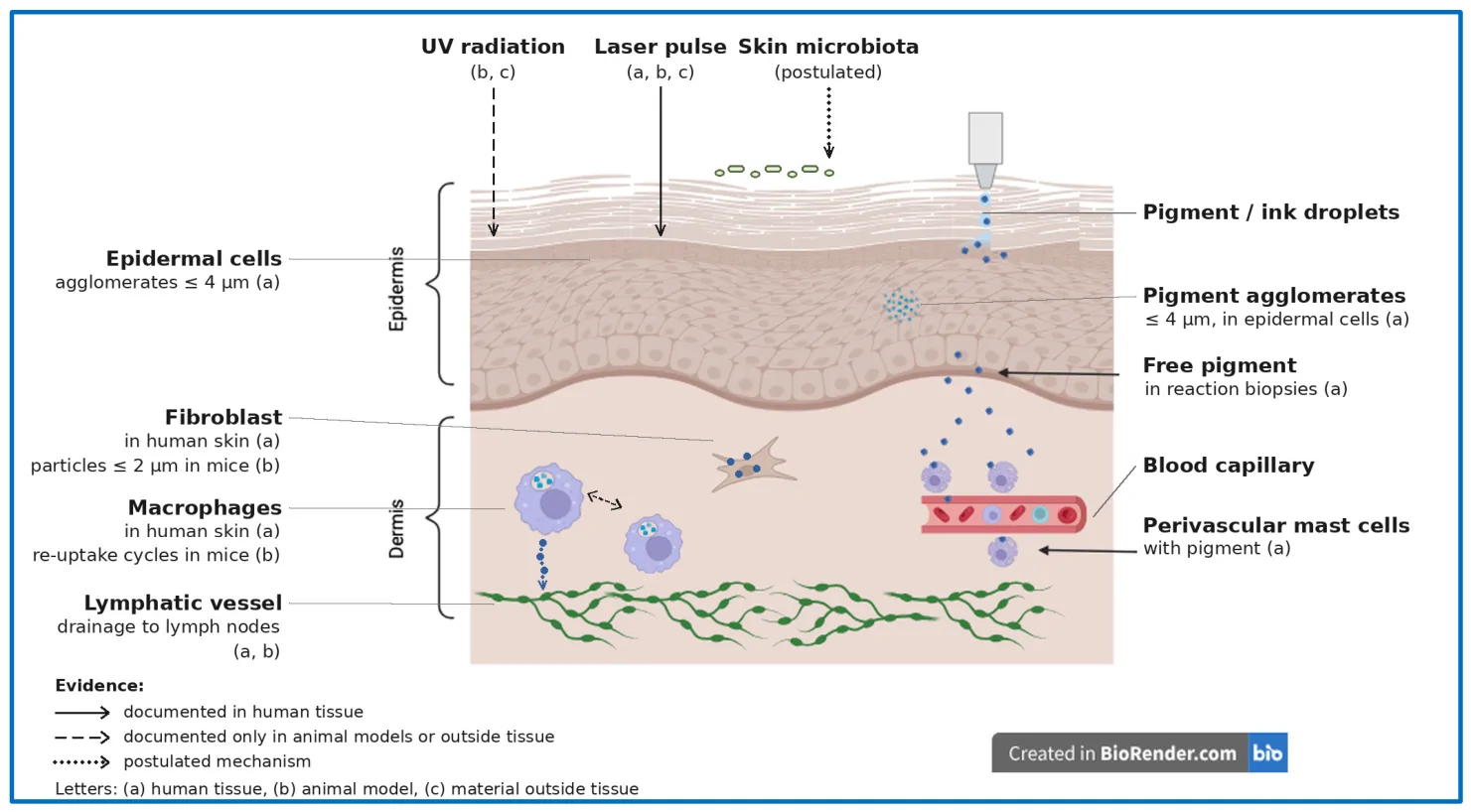
Cellular localisation of tattoo pigment in the skin and the routes of its transformation and transport. Line styles and letters indicate the evidence underlying each element, using the categories defined in Section 2: (a) human tissue, (b) animal model, (c) material outside tissue. Solid arrows denote effects documented in human tissue, dashed arrows effects documented only in animal models or outside tissue, and dotted arrows postulated mechanisms; arrows and thin lines attached to labels only identify structures. Blue dots represent pigment particles and ink droplets; the grey device in the upper right corner represents the tattoo needle; the green ovals on the skin surface represent skin bacteria. Colours and shapes only distinguish structures and cell types and carry no information on the type of evidence, which is indicated by line style and letters alone. In human skin, pigment agglomerates not exceeding four micrometres have been observed in vivo in keratinocytes, dendritic cells and basal layer cells of the epidermis, and pigment in macrophages, fibroblasts and perivascular mast cells of the dermis at depths of seventy to ninety-five micrometres [14]; the fluorescence lifetime signature of these mast cells has been interpreted as activation [14,25]. The retention of pigment through cycles of release and re-uptake by successive generations of macrophages (dashed double-headed arrow) has been demonstrated only in mice [12], and the lower pigment load of fibroblasts relative to macrophages has likewise been shown only in mice [26]. Extracellular pigment granules have been described in biopsies of red tattoo reactions [27]. Pigment and elements reach the regional lymph nodes, with preferential transport of smaller particles, as documented post mortem in human tissue [11] and in a porcine model [18]; the route by which pigment released from macrophages would reach the lymphatic vessel (dotted arrow beneath the macrophage) is postulated. The laser pulse fragments pigment particles in human skin [28,29], whereas chemical cleavage products have been identified in pigment suspensions, porcine skin samples and a mouse model [3,4,19,30]. Photodegradation by ultraviolet radiation has been demonstrated outside tissue and inferred from light-induced pigment loss in mice (Section 3.9). Reduction of the azo bond by skin bacteria has been shown only for soluble azo dyes in culture [31] and is postulated for deposited pigment (dotted arrow). Possible lysosomal oxidation, mediator release by pigment-laden mast cells and physiological desquamation of the epidermis are not shown. The drawing is schematic and not to scale.

**Figure 2:**
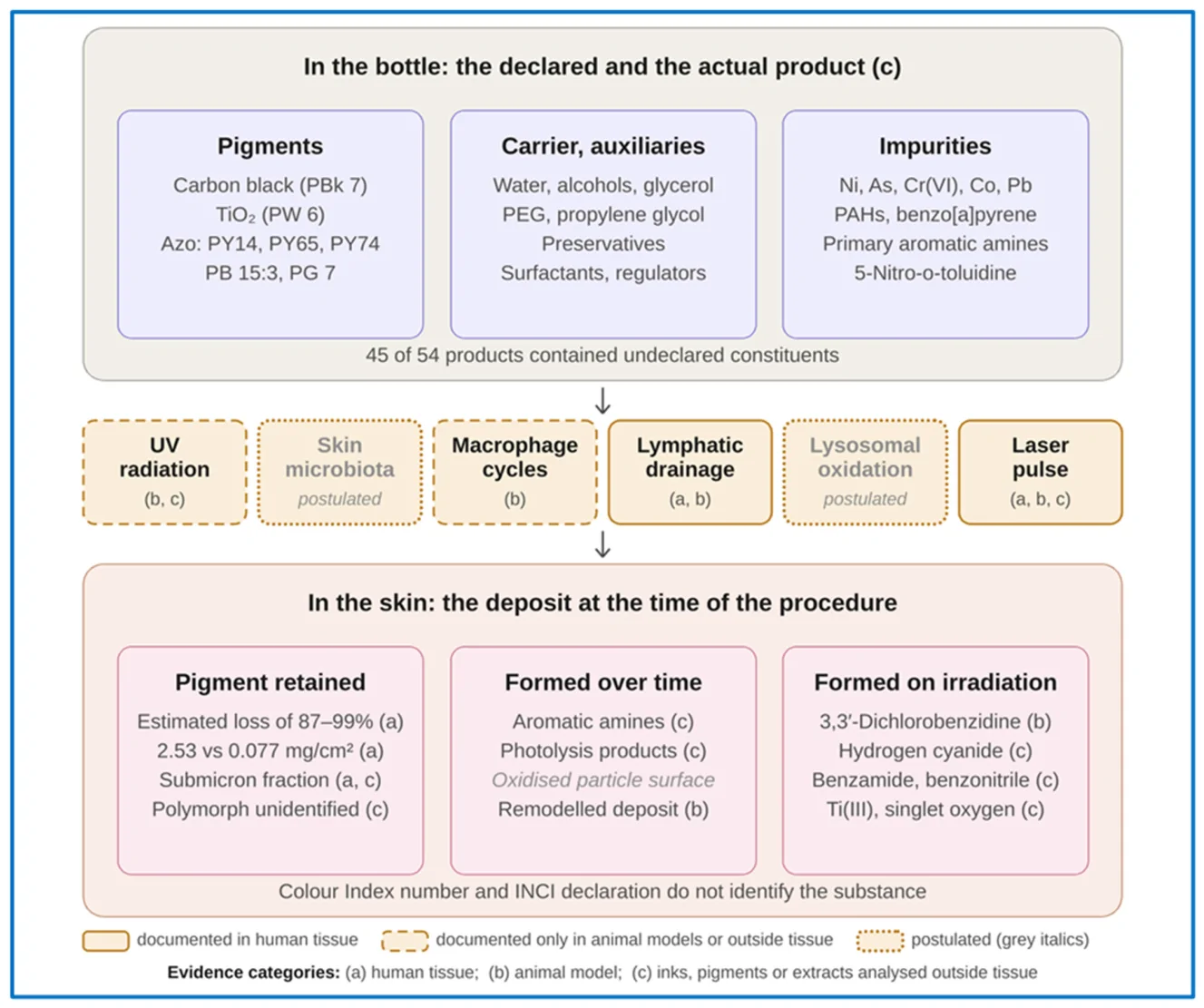
Composition of the ink before injection compared with the deposit present in the skin at the time of a removal procedure. The upper panel lists the constituents of the product identified in analyses of commercial inks: pigments, the carrier together with auxiliary substances, and impurities derived from the manufacturing process. The middle tier shows the processes acting on the deposit between injection and the procedure, followed by the laser pulse of the procedure itself; the outline of each box indicates the evidence: solid, documented in human tissue; dashed, documented only in animal models or outside tissue; dotted, postulated. The lower panel lists the pigment retained, the changes and compounds expected to arise in situ over the years, and those formed on irradiation. Letters give the evidence categories defined in Section 2: (a) human tissue, (b) animal model, (c) material outside tissue; items in grey italics are postulated. The downward arrows indicate the chronological order, from the product before injection to the deposit at the time of the procedure. The colours only distinguish the product (upper panel), the processes (middle tier) and the deposit (lower panel); neither the arrows nor the colours carry information on the type of evidence, which is indicated by box outlines, letters and grey italics alone. The estimated loss of 87–99% was derived by comparing concentrations measured in different specimens, immediately after ex vivo tattooing [20] and in skin tattooed years earlier [21], not by following the same tattoo over time (Section 3.5).

No summary quality score or risk-of-bias rating was assigned. Established risk-of-bias instruments were developed for clinical, observational, diagnostic or animal intervention studies, and none of them is suited to the heterogeneous evidence assembled here, which consists predominantly of analytical characterisations of products, imaging of pigment localisation, animal models of pigment retention and regulatory documents. Each publication in the core synthesis was instead characterised by the directness of its evidence with respect to the object of the review, namely the pigment deposit in human skin, using five categories: (a) human tissue studied in vivo, in biopsies, post mortem or in excised skin; (b) animal models; (c) inks, pigments or extracts analysed outside tissue, including suspensions, solutions and cell cultures; (d) secondary sources, that is, systematic reviews, surveys of studies and scientific reports; and (e) normative and enforcement documents. The material studied, the size of the sample and the principal constraint on inference were recorded for each publication (Appendix A, Table A2). This characterisation governed the strength of the statements made in the synthesis: statements on the composition of commercial inks rest on product analyses and market surveillance; statements on the deposit in human skin rest on category (a) wherever such data exist; and findings obtained only in animal models or outside tissue are identified as such wherever they are cited in Section 3, Section 4 and Section 5. Interpretations that extend beyond the material in which the underlying observation was made, including proposed mechanisms of transformation, transport and toxicity, are explicitly labelled as hypotheses. The same convention is applied in Figure 1 and Figure 2, in which effects documented in human tissue, effects documented only in animal models or outside tissue, and postulated mechanisms are distinguished by line style and marked with the evidence categories defined above.

Core synthesis and contextual literature

The references cited in the review fall into three groups. The core synthesis comprises the publications and documents that met all three eligibility criteria (Appendix A, Table A2); three of them [19,23,28] were assigned to this group on account of data obtained before irradiation or in untreated tissue. Studies of laser irradiation and of clinical tattoo removal [3,4,29,32,33] do not meet the second criterion, which is confined to the deposit in the absence of any removal procedure, and are cited only to frame the problem and to derive implications for laser treatment. The remaining references constitute contextual background: epidemiological studies of tattooing, tattoo removal and tattoo-related skin reactions [1,2,15,17,34]; regulatory commentary, narrative reviews and commentaries [6,35,36,37]; methodological and terminological sources [13,25,38]; clinical reports and a systematic review of cutaneous tumours arising within tattoos [39,40,41]; and general chemical, microbiological and pharmacological references [31,42,43,44]. Contextual references were not subject to the eligibility criteria and were not used as evidence for the characterisation of the deposit.

Data synthesis. Given the methodological heterogeneity of the material retrieved, which spans analytical spectrometry, in vivo and ex vivo imaging, animal models of pigment retention and regulatory market surveys, quantitative synthesis and formal meta-analysis were not applicable. The findings from the publications in the core synthesis were therefore synthesised narratively into a mechanistic framework covering pigment taxonomy, particle characteristics, baseline impurities, cellular dynamics and their implications for laser therapy, the latter drawing also on the laser-irradiation and clinical studies identified above; within this framework, empirical findings are distinguished from the mechanistic hypotheses derived from them, in accordance with the appraisal of the evidence.

## 3. Results

### 3.1. Chemical Taxonomy of Pigments

In the chemistry of colouring agents, the division into pigments and dyes does not refer to molecular structure but to the behaviour of the substance towards the medium in which it is applied [38]. A pigment is a colouring agent that is practically insoluble in that medium and remains within it as a dispersed solid phase; a dye dissolves in it, forming a system that is homogeneous at the molecular level. The distinction is therefore operational rather than structural, which means that the same substance may behave as a dye in one medium and as a pigment in another. The superordinate term covering both classes is colourant [6,35].

For the dermis as the medium of application, this distinction defines two mutually exclusive kinetic regimes. Particulate material undergoes phagocytosis, lysosomal sequestration and size-dependent lymphatic transport, and its fate is determined by size distribution, aggregation state and specific surface area [11,45]. Material in molecular solution undergoes diffusion, protein binding, enzymatic transformation and renal or hepatic excretion, and its fate is determined by concentration, partition coefficient and enzyme affinity [42]. Inferences appropriate to one regime do not carry over to the other. This division is a conceptual framework derived from general principles of particle and solute disposition rather than a result of studies comparing the two regimes for tattoo colourants; for particulate pigment, only some of its elements have been observed directly in human skin, namely intracellular localisation in vivo [14], enclosure in membrane-bound granules on electron microscopy [28] and size-dependent transport to the regional lymph nodes post mortem [11].

Professional tattoo inks are dominated by insoluble pigments, as demonstrated by analyses of product compositions in the EU market [24] and by measurements of particle characteristics in commercial products, with black pigments falling almost entirely within the nanoparticle range [45]. The presence of particulate material in tissue after complete healing has been directly confirmed by cellular-level imaging of human skin [14,46].

Ink, however, is not identical to pigment. It constitutes a finished mixture comprising one or several pigments, a liquid carrier, auxiliary substances such as preservatives, surfactants, and viscosity and pH regulators, and impurities derived from the manufacturing process, including metals, polycyclic aromatic hydrocarbons and primary aromatic amines [24,35,47]. The actual composition may moreover diverge from the declared one: in an analysis of 54 products from the US market, 45 contained additives or pigments not listed on the label [8].

The terminological consequences of this distinction are systematically overlooked in the clinical literature, and the error occurs in two opposite directions. The first consists in attributing to a solid particle properties characteristic of a solution, that is, in inferring absorption, distribution and excretion from the solubility of the parent substance, whereas the fate of the deposit is determined by particle geometry rather than by the solubility of the chromophore. The second consists in attributing to a mixture the properties of a single component, that is, in treating an ink as though it were a homogeneous substance described by a single Colour Index number, whereas its biological behaviour arises from the entire formulation, including the carrier, the auxiliary substances and the impurities. Table 3 summarises the most commonly used pigment groups by colour, indicating their typical representatives, particle characteristics, and the biological consequences that follow.

### 3.2. Physicochemistry of the Particle

On general physicochemical grounds, particle size can be expected to influence reactive surface area, aggregation, phagocytosis, lymphatic transport, interaction with lysosomes, generation of reactive oxygen species and the potential release of chemical constituents. For tattoo pigments in human tissue, the preferential transport of smaller particles to the regional lymph nodes [11] is the only one of these relationships documented in the studies reviewed here, whereas the others are inferred from particle properties. A primary particle, an aggregate, an agglomerate and a fragment formed after laser exposure are not the same biological object and cannot be described by a single figure [11].

In a study of stock inks in general use, black pigments were the smallest and fell almost entirely within the definition of nanoparticles, white pigments were the largest, and coloured pigments occupied an intermediate position [45]. Imaging of tattooed human skin from a single donor and of cultured dermal fibroblasts exposed to diluted ink confirmed the presence of structures in the nanometre range and of their agglomerates [46].

The minimum set of parameters that should be reported in every study concerning a tattoo pigment comprises the size distribution of primary particles, the degree and type of aggregation, the specific surface area (BET or equivalent), the zeta potential, the elemental composition, and identification of the chromophore by a method independent of the manufacturer’s declaration. Such a set is difficult to find in the studies cited in the present review. The last of these requirements is met by pyrolysis coupled with gas chromatography and mass spectrometry: the method assigns a pigment to a chemical structure independently of the label and simultaneously predicts the profile of its thermal decomposition products [47,48]; its use as a proxy for decomposition on laser irradiation rests on the correspondence observed for copper phthalocyanine irradiated in aqueous suspension [3].

### 3.3. The Submicron Fraction Exists Before Any Procedure

Not every particle of nanometre dimensions is a product of laser interaction, and disregard of this distinction remains the source of a substantial part of the erroneous conclusions drawn in the literature to date. The demonstration by Høgsberg et al. [45] that black inks in commercial form fall almost entirely within the nanometre range shifts the centre of gravity of interpretation: the submicron fraction need not arise in the skin, because it is already present in the product and is introduced into the skin at the moment the tattoo is made, irrespective of whether further size reduction occurs in tissue. It follows that the mere presence of nanoparticles in the skin of a tattooed person does not constitute evidence of a previous removal procedure. By the same token, their presence in a regional lymph node does not demonstrate laser fragmentation, since the preferential transport of the smallest particles to the regional lymph nodes has also been documented in post-mortem samples from tattooed donors in whom no laser treatment was reported [11]. The decisive criterion cannot therefore be the detection of nanoparticles as such, but the demonstration of a change in their size distribution, composition or localisation relative to the baseline state.

This leads to a fundamental methodological requirement: every study comparing the state before and after a procedure must have its own baseline measurement and cannot rest on the tacit assumption that an untreated tattoo contains only particles of micrometre dimensions [11]. In practice, this means that the appropriate reference point is not untattooed skin but an untreated tattoo in the same person.

### 3.4. Non-Pigment Constituents

An analysis of 54 commercial inks available on the US market, originating from nine manufacturers, revealed the presence of additives or pigments not declared on the label in 45 products [8]. A systematic toxicological review has covered ink composition, polycyclic aromatic hydrocarbons, primary aromatic amines, metal content, the fate of pigments in the skin and the applicable regulatory frameworks [47]. Quantitative data collected on the EU market after the REACH restriction entered into force indicate that the permitted limits were exceeded for nickel in 24 of the 41 inks examined, for arsenic in 20 and for chromium (VI), estimated as 1% of total chromium, in 16, and that in several products the lifetime cancer risk modelled for assumed tattooing scenarios exceeded 1 × 10^−4^ for nickel [7]. These estimates rest on assumed ink consumption and on dermal absorption factors that, as the authors acknowledge, may not reflect chronic intradermal exposure; they are modelled estimates, not clinical outcomes observed in tattooed individuals [7].

From the perspective of the course of the procedure, it is important that metallic impurities and polycyclic aromatic hydrocarbons are likely to remain in the skin together with the pigment and to be subject to the action of the same energy pulse. Co-transport of trace elements to the regional lymph nodes has moreover been documented in post-mortem human tissue and in a porcine model [11,18]. For elements, retention in the skin is supported by the same studies, whereas no primary tissue data on polycyclic aromatic hydrocarbons were included in this review, so that their retention is inferred from their presence in the product.

### 3.5. Aromatic Amines Present in the Ink Before Irradiation

Attributing the products of pigment decomposition to the action of the laser pulse presupposes that these compounds were not present before exposure, and hence that the procedure marks the moment at which exposure begins. This assumption is empirical, is open to testing and has been tested, although the result was reported as an additional finding of the study that produced it. In the study by Vasold et al. [19], two commonly used azo pigments were laser-irradiated in suspension, and the decomposition products were quantified by high-performance liquid chromatography coupled with mass spectrometry. Irradiation led to increased concentrations of 2-methyl-5-nitroaniline, 2,5-dichloroaniline and 4-nitrotoluene, that is, of compounds with documented toxic and, in part, carcinogenic activity. The finding of decisive importance for the interpretation of these results is, however, incidental to the study’s principal thesis: the same compounds were detected in the starting material before exposure to laser light. The laser, therefore, did not introduce them into the suspension but raised their concentration in a system in which they were already present, a result obtained in vitro for which no corresponding before-and-after measurement in human skin is available among the studies reviewed. This distinction is at times blurred in the secondary literature, where the products of decomposition are attributed entirely to the laser’s action.

Where aromatic amines are present in the ink as a manufacturing impurity, the patient’s exposure to them does not begin on the day of the procedure but may continue throughout the period for which the tattoo is held; this is an inference from product analyses, since none of the studies cited has measured the release of these impurities from the deposit in human skin. Laser tattoo removal may, in such a case, add to a pre-existing exposure to compounds with a documented chemical hazard, as suggested by the increase in their concentration observed on irradiation in suspension [19]; whether this increment is clinically relevant has not been established in the studies reviewed here. The amount of azo pigment potentially involved can be approximated only indirectly: immediately after experimental tattooing of excised human and porcine skin with Pigment Red 22 under various conditions, the pigment concentration ranged from 0.60 to 9.42 mg/cm^2^, with a mean of 2.53 mg/cm^2^, depending on the size of the pigment crystals, the pigment concentration applied and the tattooing procedure [20], whereas in the five of nine specimens of skin tattooed years earlier in which red azo pigments could be identified and quantified, the mean concentration was 0.077 ± 0.046 mg/cm^2^, from which a loss of the order of 87–99% was estimated [21].

Both figures rest on assumptions that need to be stated explicitly. The loss of 87–99% is not a longitudinal measurement but a comparison between two independent data sets obtained in different materials: the range corresponds to relating the mean concentration found years after tattooing to the lowest and to the highest concentration measured ex vivo (1 − 0.077/0.60 ≈ 0.87; 1 − 0.077/9.42 ≈ 0.99). The estimate therefore assumes that the initial loading of the long-standing tattoos, which contained red azo pigments such as Pigment Red 22 or Pigment Red 112, lay within the range measured ex vivo for Pigment Red 22, that experimental tattooing of excised skin reproduces the pigment density achieved in vivo, and that extraction recovery is comparable in fresh and in long-standing deposits. The range reflects neither the dispersion of the values measured years later, nor the four specimens in which no red pigment could be identified and quantified, nor the time elapsed since tattooing. The figure of more than 760 mg, given by the source authors for a single tattoo, which they describe as frequently covering more than 300 cm^2^ [21], corresponds to the mean initial density multiplied by that area (2.53 mg/cm^2^ × 300 cm^2^ ≈ 760 mg); it additionally assumes that the whole area is filled uniformly with azo pigment at that density and should therefore be read as an illustrative order of magnitude rather than as a measured dose. With these reservations, the data are consistent with the conclusion of the source authors that the major part of the azo pigment introduced into the skin either decomposes in situ or migrates within the body [21], by routes that do not require laser treatment (Section 3.9).

In a screening study of 86 ink samples available on the European market, conducted by LC-QqQ-MS covering forty substances of high concern, 26 samples were in breach of the Resolution ResAP(2008)1 then in force [49]. Particular attention is due to the identity of the most frequently detected compound. 5-Nitro-o-toluidine, a synonym of 2-methyl-5-nitroaniline, is the same compound that Vasold et al. had determined in unirradiated material eighteen years earlier; it was found in 16 samples at a mean concentration of 29 µg/g, in every case at a level that the authors assessed as corresponding to an unacceptable health risk; this judgement was derived from concentrations measured in the products, not from outcomes observed in tattooed individuals. Breaches most often concerned red and brown inks, and counterfeit products of reduced quality were identified among the products examined [49]. An earlier Italian surveillance programme had found that about 40% of the monitored inks did not comply with Resolution ResAP(2008)1 [50].

Manufacturing impurities are moreover not confined to aromatic amines: soot-based black inks contain polycyclic aromatic hydrocarbons, several of which have high quantum yields of singlet oxygen; extracts of inks containing them reduced the mitochondrial activity of keratinocytes in culture after UVA exposure [51], whereas the generation of singlet oxygen within the dermis was proposed but not measured. Amines, therefore, represent not an exception but an instance of a broader regularity, in which an industrial colourant is introduced into the skin, accompanied by material not covered by the composition declaration.

Treating the laser and sunlight as two competing sources of aromatic amines overlooks a third route, independent of both. The azo bond undergoes reductive cleavage, and the products of this reaction are precisely aromatic amines [43]. The capacity to carry out this reaction has been demonstrated in vitro for bacteria of the human skin microbiota: in a study of 26 bacterial species, skin bacteria of the genera Staphylococcus, Corynebacterium, Micrococcus, Dermacoccus, and Kocuria reduced the soluble azo dye methyl red by 74–100% within 24 h [31].

This suggests that in tattooed skin aromatic amines could be formed without the involvement of a laser, without exposure to light and independently of the initial content of the ink. The evidence, however, concerns soluble azo dyes reduced by bacteria in culture and, in line with the distinction drawn in Section 3.1, cannot be transferred directly to insoluble pigments deposited in the dermis. Given that knowledge of the absorption, distribution, metabolism, and excretion of tattoo colourants remains fragmentary [36], this route has not yet been quantified. It could nevertheless contribute to the observed loss of pigment from the skin, which has not been apportioned between transport and decomposition in human skin [21].

In the skin of a person presenting for tattoo removal, aromatic amines may therefore originate from at least three independent sources: a manufacturing impurity present in the ink from the moment of injection, photodegradation under ordinary light exposure, and microbial and metabolic reduction of the azo bond. The laser pulse constitutes a fourth source, added to the preceding three. Any study that determines amines only after the procedure and attributes them to the procedure, in fact, attributes to it the sum of these four components. The evidence for these sources is, however, of unequal strength, and none of them has been quantified in human skin in the studies reviewed: the impurity route rests on analyses of commercial inks [19,49], laser-induced cleavage on the irradiation of pigment suspensions and porcine skin samples [4,19] and on a mouse model (Section 3.9), photodegradation on experiments outside tissue and on light-induced pigment loss in the same mouse model (Section 3.9), and microbial reduction on a single study of soluble azo dyes in bacterial culture [31], while metabolic reduction is inferred from the general chemistry of azo compounds [43]. The four-source scheme is therefore a working hypothesis that specifies what a baseline measurement must control for, rather than an established apportionment of exposure.

### 3.6. Dose

The surface density of pigment in the skin, commonly referred to as pigment loading and usually expressed in milligrams per square centimetre, is a frequently cited parameter in the literature, although subsequent citations rarely indicate whether the original value is derived from an analytical measurement or an estimate. Three studies remain the point of departure: an extraction method enabling quantitative recovery of pigment from human skin with HPLC-DAD detection [22], the quantification of pigment in excised human and porcine skin immediately after experimental tattooing, which yielded the frequently cited mean of 2.53 mg/cm^2^ [20], and quantification in vivo in a mouse model [30].

This measure is nevertheless insufficient as a description of exposure. If the biological effect scales with the reactive surface area of the particles rather than their mass, then the same mass may correspond to exposures that differ by an order of magnitude, depending on the size distribution and the degree of aggregation of the deposit. Indirect support, obtained outside tissue, comes from the observation that irradiated dried inks generate chromatographic signals of higher intensity than the corresponding pure reference pigments, which the authors hypothetically attributed to the greater specific surface area of the particles in the ink compared with agglomerated pigment powder [32]. If this assumption holds, a complete description of dose requires that surface density be supplemented by the specific surface area of the particles expressed per unit volume of tissue.

### 3.7. Cellular Localisation

In the human epidermis, pigment has been detected in vivo in keratinocytes, basal cells and dendritic cells, including in tattoos up to nine years old [14], whereas in electron micrographs of human biopsies ink particles were confined to dermal fibroblasts once healing was complete [52]. In the dermis, it has been localised in vivo in human macrophages, fibroblasts and perivascular mast cells [14] and, on electron microscopy of human tattoos, in membrane-bound granules predominantly within fibroblasts and macrophages [28]; in mice, it is taken up primarily by macrophages [12] and by fibroblasts [26,53]. The persistence of a tattoo may not result solely from the longevity of an individual macrophage, since, as shown in a mouse model, the pigment undergoes cycles of release, re-uptake and storage by successive generations of cells; whether this model applies to humans remains to be determined [12].

The model previously accepted assumed that, once introduced into the skin, pigment particles and their agglomerates are taken up by macrophages, which subsequently migrate to perivascular regions, while the contribution of other cell types remains marginal. It has been shown in mice that dermal macrophages capture pigment and retain it until the end of their own life cycle [12], and that dermal fibroblasts also take up pigment particles, albeit in far smaller amounts per cell than macrophages [26]. Earlier work in mice had confirmed the capacity of fibroblasts, in addition to macrophages, to take up and store ink particles for extended periods [53].

Intravital imaging of human skin has substantially extended this picture. In clinically fully healed skin, carbon black particles were localised within three cell types of the papillary dermis, at depths of seventy to ninety-five micrometres [14]. The first of these are macrophages measuring ten to eighteen micrometres, irregular in shape and with short autofluorescence lifetimes characteristic of a phagocytosing phenotype. The carbon black load was substantial enough that achieving the same photon density at a depth of eighty-two micrometres required approximately half the laser power needed in untattooed skin, which constitutes indirect but persuasive evidence of a high content of strongly fluorescent material within the cell. The second type are fibroblasts of no more than fifteen micrometres in cross-section, in which the pigment was distributed uniformly throughout the volume of the cell [14].

The third cell type is perivascular mast cells; pigment granules had occasionally been observed in mast cells by electron microscopy of untreated tattoos [28], so the novelty of this finding lies in its intravital demonstration. These cells, located in the dermal papilla and extending vertically into the papillary layer, were elongated, approximately 10 micrometres in length, and contained distinct deposits of carbon black [14]. Their autofluorescence lifetimes were shortened relative to those of mast cells cultured in vitro, which corresponds to the fluorescence lifetime signature of the activated phenotype described earlier by the same research group [25]; activation was thus inferred from imaging parameters rather than demonstrated by degranulation or mediator release. This means that the material subjected to laser fragmentation is located within, or in the immediate vicinity of, a cell capable of immediate degranulation, in a perivascular position. It can therefore be hypothesised that fragmentation of the deposit triggers degranulation and the rapid passage of mediators into the circulation, although neither process has been examined in pigment-laden mast cells in the studies reviewed.

The findings concerning the epidermis also proved unexpected. The belief that the epidermis is cleared of pigment within approximately four weeks through physiological epithelial renewal and regeneration of the dermal–epidermal barrier [52] functioned as a textbook assumption, despite earlier histological observations of carbon particles in epidermal melanocytes and basal cells, cited by Kröger et al. [14]. Intravital examination demonstrated the presence of carbon black agglomerates within keratinocytes, cells of dendritic morphology and basal layer cells in twelve of the sixteen tattoos examined; that is, in seventy-five per cent of cases, the oldest tattoo in which epidermal pigment was found was nine years old [14]. In all these cases, the skin remained free of clinical signs of inflammation and of adverse reactions reported by the participants. The size of the agglomerates in epidermal cells did not exceed four micrometres, and in a freshly applied tattoo followed prospectively, carbon black particles deposited around the incision were still visible 84 days after application, which the authors interpreted as delayed recovery of the epidermis [14].

The claim that pigment is absent from the extracellular matrix may be explained by the absence of detectable agglomerates. The lateral resolution of the applied method is below 0.36 µm and degrades further in the dermis due to scattering, so that non-agglomerated particles are observed only as diffuse, short-lifetime areas superimposed on cellular and extracellular structures [14]. Free pigment granules in tissue have, by contrast, been described explicitly in a histological study of biopsies from red tattoo reactions, that is, of reaction-selected specimens [27].

The cellular localisation of the deposit and the routes of its transformation and transport are summarised in Figure 1, in which documented findings are distinguished from postulated mechanisms.

### 3.8. Historical and Regulatory Background: The Historical Ink as a Substrate of the Procedure

The deposit currently being removed may have been introduced into the skin before entry 75 of Annex XVII to the REACH Regulation came into force [5]. As explained in Section 2, the available data do not allow the age or the regulatory origin of tattoos removed in 2026 to be estimated [16,17]. A deposit introduced before 4 January 2022 derives from a period in which most Member States were governed at most by the non-binding Resolution ResAP(2008)1 [15], and in which ink composition was not harmonised at Union level: the limits for impurities set out in that resolution were recommendations, and national legislation on ink composition had been adopted in only seven Member States [5]. The restriction introduced in 2022 concerns the moment a product is placed on the market and does not alter the composition of the material present in the tissues of people who were tattooed earlier.

In pre-harmonisation inks, four groups of constituents give rise to the principal hazard concerns.

Azo pigments and the aromatic amines released from them. The azo group (–N=N–) constitutes the chromophore of most yellow, orange and red pigments and at the same time the weakest bond in the molecule, susceptible to thermal, photochemical and reductive cleavage. Aromatic amines occur in ink in two independent forms: as a manufacturing impurity present from the moment of production, and as a product of azo bond cleavage. Diarylide pigments are of particular importance, since laser irradiation of Pigment Orange 13 in tattooed porcine skin samples released 3,3′-dichlorobenzidine, a compound that induced DNA strand breaks in cultured human skin cells [4].

The group comprises monoazo pigments (PY74, PY65), diarylide pigments (PY14, PO13) and naphthol reds, and in practice the entire range of warm colours: yellow, orange, red, brown and flesh tones. For permanent make-up it is the principal group, since pigments for the eyebrows, the lips and areola pigmentation rely almost exclusively on warm chromophores, usually in a mixture with TiO_2_ as a lightener.

Metallic impurities. Nickel, arsenic, chromium (VI), cobalt and lead derive from pigment raw materials and from the manufacturing process rather than from deliberate addition. Before harmonisation, limits for them had only been recommended in the non-binding Resolution ResAP(2008)1 [15], and market studies show that exceedances persist even after the introduction of harmonised limits [7].

This is the only group that shows no strict colour selectivity, since metals occur independently of chromophore class, albeit with a discernible elemental concentration: chromium in green pigments, cobalt in blue ones, iron in browns and flesh tones, and nickel across the entire range. It therefore potentially concerns every deposit. Co-transport of elements to the regional lymph nodes has moreover been documented independently of any intervention [11,18].

Polycyclic aromatic hydrocarbons in black inks. The carbon black forming the basis of black pigments is produced by incomplete combustion and contains PAHs adsorbed on the particle surface. Benzo[a]pyrene is currently subject to a limit of 0.005 mg/kg [5]; previously, the same value had only been recommended in the non-binding Resolution ResAP(2008)1 [15]. Because these compounds are surface-bound rather than incorporated into the structure of the pigment, their release, which has not been measured in tissue in the studies reviewed, would not require decomposition of the chromophore.

The group comprises all deposits based on carbon black (Pigment Black 7): black and grey tattoos, the outlines of coloured tattoos, eyeliner, eyebrows in cool shades and scalp micropigmentation. Since a black outline is present in most multicoloured tattoos, the scope of this group is in practice the broadest of the four. Black pigments, moreover, show the smallest particle size and the highest proportion of the nanometre fraction [45], so that the PAHs can be expected to be bound to the fraction with the largest specific surface area and, judging from the preferential lymphatic transport of smaller particles in post-mortem human tissue [11], the greatest mobility in tissue.

Pigments restricted on expiry of the derogation. Pigment Blue 15:3 and Pigment Green 7, that is, copper phthalocyanine and its polychlorinated derivative, have been subject to the restriction since the derogation expired on 4 January 2023 [5]; they may therefore be present in some blue and green tattoos made before that date, but their presence in any particular deposit cannot be assumed. The date on which the restriction took effect does not, however, determine the composition of a particular deposit: according to comments submitted during the restriction procedure, Pigment Green 7 had already been largely replaced by the brominated Pigment Green 36 [5], and the pigment actually present can be established only analytically. The restriction of both pigments was precautionary: they belong to the 21 colourants prohibited in hair dye products under Regulation (EC) No 1223/2009 on cosmetic products, for the majority of which the Committee for Risk Assessment of the European Chemicals Agency considered that a risk of cancer and possible non-carcinogenic hazards could not be ruled out, primarily because adequate information on their hazard properties and on the risk to human health was lacking; at the time, Pigment Blue 15:3 remained permitted in other cosmetic products and Pigment Green 7 in cosmetic products other than eye products [5]. For Pigment Blue 15:3, the formation of hydrogen cyanide following ruby laser irradiation of the pigment in aqueous suspension has been documented [3]. The macrocyclic structure with a central copper atom, responsible for high photostability in daylight, becomes, under ruby laser irradiation, a source of fragments with a toxicological profile different from that of the parent compound, whereas decomposition under Nd:YAG laser irradiation at 1064 and 532 nm was only low [3].

Beyond blue and green tattoos, the group covers violet and turquoise shades obtained through mixing, the muting agents used to modify blacks, and the cool correctors used in permanent make-up to neutralise eyebrows that have shifted towards warm tones. A corrective deposit is, by definition, applied over an earlier one, so that phthalocyanine, an azo pigment and TiO_2_ may coexist at a single anatomical site, each with a different absorption profile and a different profile of degradation products.

Combined scope. The four groups above cover the entire colour space used in tattooing and permanent make-up: warm colours fall to azo chromophores, cool colours to phthalocyanines, and black and grey to carbon black, while metallic impurities occur independently of colour. They are complemented by TiO_2_, present in whites, flesh and pastel shades and as a lightener of mixtures. No class of deposit can, therefore, be assumed a priori to be free from the problem of historical composition. The summary is presented in Table 4, bearing in mind that assigning a pigment to a class on the basis of colour is indicative only and does not replace analytical confirmation, and that the entries denote hazards or effects associated with a pigment class rather than clinical risks established for a given deposit.

The assumption of full compositional compliance is not warranted for tattoos made at present either: undeclared constituents and exceedances of metal limits have been documented in current products (Section 3.4), and counterfeit products, which can be distinguished from originals by pyrolysis–gas chromatography/mass spectrometry [54], have been identified in market screening [49].

#### Titanium Dioxide

Titanium dioxide is the principal constituent of white and flesh-toned pigments, but at the same time a widely used lightener of coloured inks: blue, yellow, green and violet [23,29]. Its presence is therefore not confined to permanent make-up in light shades and cannot be predicted from the colour of the deposit. In a series of 20 biopsied laser-treated tattoos, titanium dioxide was over-represented in those that responded poorly to treatment, although its causal role has not been established [29]; a plausible mechanism of paradoxical darkening is the reduction of Ti^4+^ to Ti^3+^, which has been demonstrated for TiO_2_ outside tissue [44] but not in tattooed skin; a colour change from white to bluish-black was reproduced by Q-switched Nd:YAG laser irradiation of a cream containing 5% TiO_2_ [29]. As a photocatalytic material it can modify the course of photodegradation of the organic pigments present in the same mixture. From the standpoint of laser therapy, it is therefore not an inert filler but an active participant in the reaction. Experimental support, obtained outside tissue, comes from the study of Aljubran et al. [32], in which dry yellow pigments (PY14, PY65, PY74) and their mixtures with rutile TiO_2_ (50:50 by weight), as well as dried commercial inks, were irradiated with a QS Nd:YAG 532 nm laser in glass vials and characterised by headspace GC-MS, SEM-EDX, DLS and XRD. Unirradiated samples generated no chromatographic signals. Pigment irradiated on its own underwent fragmentation (PY14: from 705 to 301 nm), whereas the same pigment in a mixture with TiO_2_ agglomerated, reaching approximately 460 nm, close to the size of irradiated TiO_2_ alone (approximately 470 nm), rather than that of the fragmented pigment. The authors proposed two cooperating mechanisms, neither of which was measured directly. The first was optical, with TiO_2_ absorbing or reflecting the laser light, thereby reducing the energy available to the chromophore; the second was thermal, with particles fusing rather than decomposing at local temperatures above 1000 °C reported in the laser literature rather than measured in the study. SEM imaging showed direct contact between TiO_2_ particles and the pigment surface.

What proved decisive, however, was a change in the chemical profile rather than its mere reduction. In the mixtures with TiO_2_, the fragments recorded in pure PY14 disappeared, but at the same time a signal absent from it appeared, tentatively assigned to benzamide or benzonitrile. Under these conditions, titanium dioxide therefore did not simply suppress degradation but redirected it, generating products not observed in its absence. The effect moreover appeared to be graded and dependent on TiO_2_ content, although this inference rests on a small number of commercial inks rather than on a controlled series of TiO_2_ concentrations: in an ink declaring 25%, the smallest increase in particle size after irradiation was recorded, together with a substantial reduction in peak intensity relative to an ink without TiO_2_ of almost identical declared composition.

### 3.9. Chemical Ageing of the Pigment In Situ

The clinical literature tacitly assumes that pigment deposited in the skin remains an inert system and that the only chemical intervention in its structure is the laser pulse. This assumption cannot be maintained in the light of the available data: over the years the deposit may be subject to as many as five concurrent modifying processes, documented to differing degrees, encompassing both chemical transformation of the molecule and spatial and quantitative remodelling of the deposit as a whole. Of these processes, only lymphatic transport has been documented in human tissue [11]; photodegradation has been demonstrated outside tissue, remodelling through cycles of macrophage uptake has been shown in mice [12], metabolic transformation is poorly characterised, and lysosomal oxidation remains a hypothesis.

The best documented of these is photodegradation induced by ultraviolet radiation. The decomposition of organic pigments under sunlight and under simulated radiation has been demonstrated directly in vitro, with azo pigments decomposing into numerous products, whereas phthalocyanines, quinacridones and dioxazines proved relatively photostable under the conditions applied [55]; for Pigment Yellow 74 the course of photodecomposition has been described in detail in tetrahydrofuran solution [56]. Metabolic transformations remain considerably less well characterised: only two studies address the metabolism of tattoo pigments, as against eleven devoted to photolysis [57]. To this may be added oxidation and modification of the particle surface, occurring among other places in the lysosomal compartment of the macrophage, that is, in an environment of low pH and high oxidative activity, although no study cited here has demonstrated this process directly for tattoo pigments. The two remaining processes do not alter the chemical structure of the chromophore but the distribution of the material in the tissue. Cycles of uptake and re-uptake by successive generations of macrophages lead, in mice, to spatial remodelling of the deposit without macroscopic change of the tattoo [12], whereas lymphatic transport, documented by the presence of pigment in the regional lymph nodes of tattooed donors post mortem [11], is likely to contribute to its gradual loss, although its time course has not been measured in humans. Quantitative in vivo data on both mechanisms were obtained in an animal model by Engel et al. [30]: in SKH-1 mice, the amount of Pigment Red 22 in the skin decreased by about 32% within 42 days of tattooing; laser exposure reduced it by about 51% compared with non-irradiated skin, part of this reduction being attributed to photodecomposition on the basis of the detection of the corresponding aromatic amines; and 32 days of simulated solar radiation reduced it by about 60%, a loss that the authors assumed to reflect decomposition in the skin. The epidemiological pattern is consistent with this picture, although it does not in itself prove it: in a Danish population study of 5914 tattooed individuals, skin reactions persisting beyond the first three weeks after tattooing were reported by 10.2% of respondents, and having held a tattoo for more than ten years was associated with almost threefold higher odds of a reaction (adjusted odds ratio 2.92; 95% CI 1.45–5.88), while coloured tattoos carried higher odds than black ones [34]. The dependence on the length of time for which a tattoo has been held is difficult to reconcile with the assumption of an inert deposit.

It may therefore be held that the deposit at the time of the procedure is not identical with the ink in its container at the time of tattooing.

The composition of the ink before injection and that of the deposit present in the skin at the time of the procedure are compared in Figure 2, with the evidence underlying each element indicated.

## 4. Discussion

### 4.1. The Substrate of the Procedure Is Incompletely Characterised

The aim of the review was to establish what is present in the skin of a tattooed person before any removal intervention, and to assess the extent to which this material remains identifiable. The evidence assembled indicates that it remains identifiable only to a limited extent: the substrate of the removal procedure is incompletely characterised, and this uncertainty has three independent, cumulative layers. First, the declaration of composition does not reliably correspond to the contents, as documented by product analyses and market surveillance in the United States and the European Union [8,9,10]. Second, the Colour Index number and the INCI declaration do not unambiguously identify the substance: they determine neither the polymorph, nor the particle size distribution, nor surface coatings, nor accompanying impurities, while counterfeit products, which can be distinguished from originals by pyrolysis–gas chromatography/mass spectrometry [54], have been identified on the European market [49]. Third, the deposit has probably changed since the time of tattooing: the available data point to photodegradation, demonstrated outside tissue [55,56], spatial remodelling through cycles of uptake, release and re-uptake by successive generations of macrophages (demonstrated in mice) [12], and a loss of pigment estimated at 87–99% of the initial concentration in skin tattooed years earlier [20,21], an estimate whose assumptions are set out in Section 3.5; oxidation and transformation in the lysosomal compartment remain hypothetical.

An additional, conditional layer is regulatory. Tattoos made before entry 75 of Annex XVII to the REACH Regulation came into force [5] were made with inks whose composition was not harmonised at Union level, which does not in itself imply that a given deposit contains a substance restricted today. In addition, the data assembled in this review do not allow the share of such tattoos in the present-day treatment population to be determined (Section 2). Moreover, market data collected after 2022 do not confirm a complete turnover of the pigment pool [7,9]. For these reasons, studies of currently restricted pigments were not excluded on the basis of publication date (Section 2).

The incomplete characterisation of the substrate should not, however, be equated with demonstrated harm, and the evidence assembled here has to be read with the distinction between chemical hazard and clinical risk in mind. Most of this evidence establishes hazard: the presence in inks of substances now restricted under entry 75 [7,9,49] and the formation of compounds of toxicological concern from pigments irradiated under experimental conditions [3,4,19,55]. Evidence of distribution beyond the site of injection, such as the transport of particles and elements to the regional lymph nodes [11,26], documents exposure but not its consequences. In the studies cited here, health risk was assessed from concentrations measured in products, in one case combined with modelled exposure scenarios [7,49], and the Union restriction itself infers a general level of risk from the hazard classification of substances combined with the way in which the mixtures are administered [5]. None of these approaches measures the dose actually released from a deposit in human skin. The clinical outcome data cited in this review concern local effects, namely skin reactions [27,34] and paradoxical darkening or non-response after laser treatment [29], and they do not establish a causal link with a specific constituent of the deposit. For the systemic and carcinogenic hazards discussed here, including aromatic amines, polycyclic aromatic hydrocarbons, metallic impurities and hydrogen cyanide, the evidence assembled therefore documents a hazard and a plausible route of exposure, but not a demonstrated clinical risk.

A separate finding of clarifying importance is the presence of a submicron fraction in the deposit prior to any intervention [11,45]: the detection of nanoparticles in tissue or in a lymph node proves neither a previous procedure nor laser fragmentation, and only a change relative to the baseline state can be decisive (Section 3.3).

### 4.2. Implications for the Design and Interpretation of Studies on Degradation of the Deposit

Three methodological requirements follow from the findings set out above. The first is the requirement of a baseline measurement: every study comparing the state before and after a procedure must have its own baseline value, and the appropriate reference point is not untattooed skin but an untreated tattoo in the same person (Section 3.3). This requirement is of particular importance for the determination of aromatic amines, which may originate from a manufacturing impurity, from photodegradation and from reductive cleavage of the azo bond independently of the laser; the four-source scheme set out in Section 3.5 is a hypothesis that defines the confounders a baseline measurement must control, not an established apportionment of exposure.

The second requirement is the separation of the kinetic regimes of particle and solution (Section 3.1): inferences appropriate to one regime do not carry over to the other, and the properties of a single component must not be attributed to a mixture. The third requirement is an adequate measure of dose: if, as suggested by experiments outside tissue [32], the reactive surface rather than the mass of the particles governs the effect, surface density in milligrams per square centimetre should be supplemented by the specific surface area of the particles expressed per unit volume of tissue (Section 3.6).

A further practical consequence is that results obtained for a pigment in isolation cannot be transferred directly to real deposits: because titanium dioxide can redirect the laser degradation of the same chromophore outside tissue (Section Titanium Dioxide) [32], a multi-pigment deposit, typical in particular of permanent make-up and of corrective layers, constitutes a distinct object of study rather than the sum of the behaviours of its components.

### 4.3. Clinical Implications: Patient Selection and the Sequence of Methods

The incomplete characterisation of the substrate also bears on the assessment of treatment algorithms. In the study by Pióro et al. [33], a hue parameter H value above 15.3, measured before the first session, was associated with the need to supplement laser therapy with a chemical preparation (AUC = 0.703; *n* = 75). Within the protocol studied, however, the chemical preparation was added only when laser treatment alone proved insufficient, so that the indicator informs a decision taken late in the treatment pathway rather than the initial allocation of patients; used in this way, it cannot prevent therapy from beginning, in patients with high H values, with the method less likely to suffice.

The scope of this conclusion nevertheless requires precise limitation. An AUC of 0.703 corresponds to moderate discriminatory ability and does not permit any judgement as to the ineffectiveness of the laser in an individual case; the authors themselves indicate the need to supplement the model with additional variables before its clinical implementation. None of the colour parameters examined correlated with the number of sessions required, which means the model determines only whether a second method will be required, not how labour-intensive the therapy will be. The indicator should therefore be treated as a signal justifying separate allocation, rather than as a threshold that permits the outcome to be settled in advance.

The order in which the methods are applied is of fundamental importance in this context, for two independent reasons. First, a pigment feature that heralds limited laser efficacy may also herald an elevated risk of complications. Higher H values correspond to yellow and orange shades, associated with the presence of iron oxides; this is, however, an interpretation of the observed colour and not an analytical identification of the substance present in the skin. The parameter H measured photographically depends not only on the chromophore but also on the depth of the deposit, on the scattering and absorption of light in the tissue, on the presence of melanin and haemoglobin, and on the mutual masking of the components of a mixture; the shift in hue of a more deeply situated pigment towards cool colours (the Tyndall effect) renders this measure all the more indirect. The same colour phenotype may conceal an inorganic pigment, an organic yellow pigment, a mixture of several pigments, or a formulation that additionally contains TiO_2_, whose presence alone modifies the course of laser degradation. Paradoxical darkening has been described both for iron oxides and for TiO_2_ [29], and its mechanism has been hypothesised to involve the reduction of Fe^3+^ to Fe^2+^ and of Ti^4+^ to Ti^3+^ [29]; the latter reduction has been demonstrated for TiO_2_ outside tissue [44], but neither reaction has been demonstrated in tattooed skin in the studies reviewed. Darkening is not, as a rule, irreversible: in some of the cases described, subsequent laser sessions led to lightening, whereas in others surgical excision proved necessary. The appropriate characterisation is therefore a complication with an unpredictable course, which further complicates therapy, rather than permanent deterioration as an inevitable consequence.

Second, the two methods probably direct the pigment material in opposite directions. Laser therapy does not remove the colourant from the body but fragments it, as electron microscopy of laser-treated human tattoos shows [28], thereby favouring redistribution via lymphatic drainage, with part of the material remaining in dermal macrophages, as suggested by the recapture of released pigment by dermal macrophages in mice [12]; extraction methods, by contrast, are based on the assumption of transepidermal removal of the material. This claim nevertheless remains a hypothesis rather than an established fact: there are no mass-balance studies that would allow the two processes to be quantified and compared directly. This hypothesis is at the same time the best available justification for a study of the sequence of methods and the principal question that such a study should answer. A separate caveat is that a safety advantage of extraction methods cannot be assumed *a priori*: chemical removers cause local adverse effects, including tissue damage, scarring and pigmentary disturbance. A comparison of sequences must therefore encompass both the fate of the pigment material and the safety of the tissue.

For the same reason, qualification for the procedure cannot rest on the characteristics of the pigment alone, since the tattooed skin may itself harbour melanocytic lesions that require a diagnosis before any intervention. Tattoo ink can mask colour changes in such lesions [39,40] and may hinder their dermoscopic assessment, because it alters how the distribution of pigment, the vascular structures and the borders of a lesion appear [56]. The clinical report by Bettolini et al. [39] illustrates this problem. In a 42-year-old man, multiple asymptomatic brownish papules had arisen within the red band of a tattoo completed a decade earlier. Dermal aggregates of spindle-shaped melanocytes without mitotic activity and their immunohistochemical profile were considered compatible with the plexiform variant of Spitz naevus, although fluorescence in situ hybridisation for anaplastic lymphoma kinase rearrangement was inconclusive; excision of the red band showed similar findings in the remaining papules. The authors conclude that Spitz naevus should be considered in the evaluation of papular tattoo reactions [39]. Confinement to a single ink colour is not, however, a marker of benign behaviour: four nodules restricted to the red parts of a multicoloured tattoo proved to be a primary nodular melanoma with a Breslow thickness of 11 mm and three in-transit metastases [41].

Because a laser session yields no tissue specimen, a lesion included in the treatment field without prior sampling is exposed to laser energy without a diagnosis. Qualification should therefore include examination of the entire treatment area, with dermoscopy of any papular, nodular or pigmented lesion and histological verification of lesions whose nature remains uncertain. A systematic review of 160 published cases of tumours arising within tattoos, 43 of them melanomas, likewise advises biopsy of a concerning lesion, deferral of laser removal until appropriate treatment has been completed, and screening of the tattoo throughout laser treatment [40]. Photographic and dermoscopic documentation before the first session would, in addition, provide a clinical counterpart to the baseline measurement required in Section 4.2. The assessment may also determine the method itself: in the case described, excision removed the pigment while allowing the remaining papules to be examined histologically [39], which laser treatment could not have done. The evidence nevertheless rests predominantly on case reports, and the number of published cases does not allow incidence to be estimated [40]. Nor can the restriction of the lesions to the red component, which Bettolini et al. interpret as a hint of the tattoo’s possible involvement, be attributed to any ink constituent, since no analysis of the ink is reported [39]. The case thus reproduces, in a single patient, the incomplete characterisation of the substrate discussed in Section 4.1.

### 4.4. Directions for Future Research

Decisive answers could be provided by a randomised trial comparing two pathways: laser therapy as first-line treatment with chemical extraction as a rescue measure, versus a model in which extraction is the primary management and the laser serves solely to eliminate residual pigment. Stratification should take into account baseline colour parameters, the result of analytical identification, the depth and density of the deposit, and skin phototype.

The inclusion criterion should encompass the characteristics of the material currently present in the skin, rather than the documentation of the product originally used. Information about the ink originally applied does not, after years, reproduce the characteristics of the deposit: what matters are the proportions of the pigments, the size and morphology of the particles, admixtures, surface coatings and crystalline phase, while the material in the tissue may undergo photodegradation, chemical transformation and selective loss through transport to the regional lymph nodes (Section 3.9). The feasibility of such identification is, moreover, a methodological problem in its own right, requiring resolution at the protocol stage.

The primary endpoints should comprise the mass balance of the pigment material: the fraction eliminated transepidermally relative to the initial load, the material remaining locally, and the material redistributed. The analytical panel for chemical transformation products must in turn be matched to the identified composition, since there is no universal biomarker covering all classes of pigment: the determination of aromatic amines in urine has value for azo pigments but not for TiO_2_ or for iron oxides. Monitoring must, without exception, include a baseline measurement before any intervention (Section 4.2). Secondary endpoints should include the frequency of paradoxical darkening, scarring, hypopigmentation, and hyperpigmentation; inflammatory and immunological reactions; the total number of sessions; the duration of therapy; digital image analysis parameters; and patient satisfaction.

The overriding limitation of assessments of efficacy to date remains the equation of reduced pigment visibility with pigment removal. The aesthetic effect, the elimination of material from the body and biological safety constitute three distinct endpoints, and colour measurement decides only the first of them.

## 5. Conclusions

The review has enabled the formulation of findings on the chemical identity of the pigment present in the skin and its transformations over time, along with implications for clinical management and the regulation of the ink market. The evidence assembled indicates that uncertainty as to the composition of the substrate is not a marginal problem but one that conditions the interpretation of research on tattoo removal and sets the limits of any inference about the safety of the procedure.

1. The material subjected to tattoo removal remains incompletely characterised. This state results from three independent, cumulative layers of uncertainty: the declared composition does not necessarily correspond to the actual content, the Colour Index number does not unambiguously identify the substance, and the deposit has probably undergone transformation since the tattoo was made.
2. The deposit is unlikely to constitute an inert system. A loss of pigment has been estimated from human skin specimens, photodegradation has been demonstrated outside tissue and spatial remodelling in mice, whereas oxidation and lysosomal transformation remain hypothetical. Consequently, the question of its toxicity requires reformulation: instead of determining whether a given pigment was toxic, it is necessary to establish which compounds may arise as a result of its degradation, in what amounts they reach the tissues and whether exposure to them is associated with clinical outcomes, since a documented chemical hazard does not in itself demonstrate a clinical risk.
3. The date of publication cannot constitute a criterion for excluding literature on currently restricted pigments, because restrictions are not retroactive and these substances may still form part of the treatment material in the present patient population.
4. A submicron fraction is present in the deposit before any intervention, and its detection in tissue or in a lymph node therefore proves neither a previous procedure nor laser fragmentation. Every study assessing the effects of a procedure consequently requires a baseline measurement, the appropriate reference point being an untreated tattoo in the same patient.
5. In terms of clinical practice, qualification for the procedure should be based on the characteristics of the material currently present in the skin, and on a dermatological assessment of the tissue in which it lies, with histological verification of any lesion of uncertain nature before treatment begins, and the content of informed consent for a removal procedure should include information that the composition of the substrate is incompletely characterised and that the chemical hazards identified have not been translated into a quantified clinical risk, rather than being confined to local risk.
6. In regulatory terms, the obligation to confirm the identity of a pigment analytically should be assigned to the moment a product is placed on the market, and this requirement should also cover pigments introduced as substitutes for restricted substances.

## 6. Limitations of the Review

The present review is subject to several methodological and material limitations. The first of these, concerning the identifiability of substances, is at once a finding of the work and a limitation of it. Since neither the Colour Index number nor the INCI declaration identifies a substance unambiguously, the collation of data from independent studies rests in part on declarations whose reliability is called into question in the Results section. The scale of the phenomenon is illustrated by the findings of the present review: undeclared constituents were found in a substantial proportion of products; the polymorph of Pigment Blue 15 could not be unambiguously determined; and counterfeit products have been identified on the market. In cases where the source study identified the pigment solely on the basis of the label, the assignment of a result to a particular substance is therefore conditional.

The second limitation arises from the isolation of pigment in the source studies, which reduces the transferability of the results to the mixtures used in practice. A substantial part of the data on photostability and fragmentation derives from experiments conducted on suspensions of single pigments, whereas an actual deposit constitutes a mixture of pigments, carrier, auxiliary substances and impurities. Moreover, the presence of TiO_2_ alone can redirect the laser degradation of the same chromophore outside tissue (Section Titanium Dioxide). Conclusions obtained for a pigment in isolation cannot consequently be extrapolated directly to commercial formulations, nor to multi-pigment deposits, which are typical, in particular, of permanent make-up.

The third limitation is the heterogeneity of the research models, which encompass the mouse, the pig, human tissue ex vivo and intravital imaging in humans, with dose scales differing by orders of magnitude: from 0.60 to 9.42 mg/cm^2^ immediately after tattooing to a mean of 0.077 mg/cm^2^ in deposits of many years’ standing; because the former values were obtained after experimental tattooing of excised skin and the latter in other, long-standing human tattoos, the loss derived from them is a cross-sectional estimate rather than a measured change (Section 3.5). The comparability of results is further limited by the diversity of measurement techniques, since particle size determined by DLS, SEM, and synchrotron methods is not a single, uniform quantity. The minimum set of physicochemical parameters postulated in the present work is reported in the source literature only exceptionally. Moreover, several of the processes discussed, including the retention of pigment through cycles of macrophage uptake and release and the light-induced loss of pigment in vivo, have been demonstrated only in mice, and the chemical products of laser and light exposure have been identified predominantly in suspensions, dry pigment preparations, solutions and animal tissue; their extrapolation to the human deposit therefore remains to be verified.

The fourth limitation is the uneven coverage of pigment classes, which depends on the type of evidence. Among the studies reviewed, data obtained in tissue concern predominantly two classes: carbon black, the only pigment localised intravitally in human skin [14], and red azo pigments, the only class for which pigment loading, its decrease years after tattooing and its light-induced loss in a mouse model have been quantified [20,21,30]. For yellow azo pigments, copper phthalocyanines and titanium dioxide, the products of irradiation are known almost exclusively from experiments outside tissue [3,32,55,56], although phthalocyanines and TiO_2_ have been identified in human skin and regional lymph nodes post mortem [11]; data on the laser degradation of pigment mixtures, such as those typical of permanent make-up, are limited to binary pigment–TiO_2_ mixtures and dried commercial tattoo inks irradiated outside tissue [32]. The imbalance extends to the types of transformation: eleven studies on photolysis compare with two on the metabolism of tattoo pigments [57], so that conclusions concerning non-photochemical routes rest on isolated studies. It is therefore a feature of the available data rather than of the literature selection adopted in the present review; the lack of quantitative data on the decomposition products formed on laser or solar exposure was pointed out a decade ago [37].

The final group of limitations relates to the search strategy adopted. It involved a language restriction to studies in English, Polish, German, and French, a search limited to the title and abstract fields, and reliance on four bibliographic databases, which may have resulted in the omission of studies published in other languages or not indexed in those databases. Access to manufacturers’ grey literature, including composition documentation and validation data, remained limited, whereas regulatory documentation was covered only by a targeted search. In addition, the truncated term impurity* does not retrieve the plural impurities: when block A was re-run in PubMed with impurit*, the overall result set grew by four records, one of which [20] had entered the core synthesis through another route. The reproducibility of the search yields could, moreover, be verified only for PubMed, because the yields of the Embase, Scopus and Web of Science searches and the list of seed publications used for citation checking were not available for this revision.

## Figures and Tables

**Table 1:** Distribution of record membership across the thematic blocks (PubMed, 14 August 2026; *n* = 639).

Number of Blocks Whose Criteria a Record Meets	Number of Records	Share
1	542	84.8%
2	73	11.4%
3	23	3.6%
4	1	0.2%
Total in more than one block	97	15.2%

**Table 2:** Intersections of pairs of thematic blocks (number of records).

	A	B	C	D
A	151	24	28	23
B	24	151	35	18
C	28	35	321	20
D	23	18	20	138

Description: The letters A–D denote the four thematic blocks of the search strategy: A (composition, impurities and labelling), B (physicochemical characterisation of the particle), C (tissue and cellular localisation) and D (transformations occurring in situ). Each block is a group of search terms (keywords and phrases) combined with the population core using the AND operator. Diagonal values give the number of records retrieved for each block; off-diagonal values give the number of records retrieved by both blocks of the pair.

**Table 3:** Main groups of tattoo pigments and their characteristics. The last column combines observations with consequences expected from particle characteristics and chemical structure; entries described as possible have not been demonstrated in human skin.

Group	Typical Examples	Particle Features	Biological Significance
Black	Carbon black (Pigment Black 7), occasionally iron oxides	Smallest; high proportion of nanoparticles	Large reactive surface, phagocytosis, possible generation of ROS
White and flesh-toned	TiO_2_/Pigment White 6/C.I. 77891	Usually larger particles or aggregates	Persistence, lightening of mixtures, relevance for laser therapy
Red, yellow, orange	Azo pigments, quinacridones, iron oxides	Dependent on pigment and aggregation	Possible degradation to aromatic amines under defined conditions
Blue and green	Copper phthalocyanines, Pigment Blue 15:3, Pigment Green 7	Particles of intermediate size	Photostability and possible degradation products dependent on structure

Abbrevations: TiO_2_, titanium dioxide. Pigments are designated by their Colour Index (C.I.) generic names and constitution numbers.

**Table 4:** Mapping of deposit colour onto chromophore class and principal hazard concern. The hazard concerns listed derive from product analyses, regulatory assessments and experiments on pigments outside human skin, with the exception of darkening, which is a clinical observation (Section Titanium Dioxide).

Colour of the Deposit	Typical Pigment Class	Principal Hazard Concern	Typical Application in PMU
Black, grey	Carbon black (PBk 7)	PAHs; nanometre fraction	Eyeliner, cool-toned eyebrows, scalp micropigmentation
Yellow, orange, red, brown	Monoazo and diarylide pigments	Aromatic amines	Eyebrows, lips, areola
Blue, green, violet	Phthalocyanines (PB 15:3, PG 7)	Hydrogen cyanide (shown for PB 15:3 under ruby laser irradiation in suspension); pigments restricted since 2023	Cool correctors
White, flesh-toned, pastel	TiO_2_ (PW 6)	Reversal of the direction of fragmentation (shown outside tissue); darkening	Lightening of mixtures, shading
All	—	Metallic impurities	—

Abreviations: PAHs, polycyclic aromatic hydrocarbons; PB 15:3, Pigment Blue 15:3; PBk 7, Pigment Black 7; PG 7, Pigment Green 7; PMU, permanent make-up; PW 6, Pigment White 6; TiO_2_, titanium dioxide. Pigment designations are Colour Index generic names.

## Data Availability

No new data were created or analyzed in this study. Data sharing is not applicable to this article.

## Abbreviations

The following abbreviations are used in this manuscript:

AUC	area under the curve
BET	Brunauer–Emmett–Teller (method of specific surface area determination)
CI	confidence interval
C.I.	Colour Index
DLS	dynamic light scattering
DNA	deoxyribonucleic acid
EC	European Community
EU	European Union
GC-MS	gas chromatography–mass spectrometry
HPLC-DAD	high-performance liquid chromatography with diode array detection
INCI	International Nomenclature of Cosmetic Ingredients
LC-QqQ-MS	liquid chromatography–triple quadrupole mass spectrometry
MEDLINE	Medical Literature Analysis and Retrieval System Online
NCBI	National Center for Biotechnology Information
Nd:YAG	neodymium-doped yttrium aluminium garnet
PAH	polycyclic aromatic hydrocarbon
PB	Pigment Blue
PBk	Pigment Black
PG	Pigment Green
PMU	permanent make-up
PO	Pigment Orange
PRISMA	Preferred Reporting Items for Systematic Reviews and Meta-Analyses
PW	Pigment White
PY	Pigment Yellow
QS	Q-switched
REACH	Registration, Evaluation, Authorisation and Restriction of Chemicals
ResAP	Resolution of the Committee of Ministers of the Council of Europe (Partial Agreement in the Social and Public Health Field)
ROS	reactive oxygen species
SEM	scanning electron microscopy
SEM-EDX	scanning electron microscopy with energy-dispersive X-ray spectroscopy
UV	ultraviolet
XRD	X-ray diffraction

## Appendix A

**Table A1:** Search strings and number of records retrieved (last search: 14 August 2026).

Database (Platform)	Search String	Records
PubMed/MEDLINE (NCBI)	#1 (tattoo[tiab] OR tattoos[tiab] OR tattooing[tiab] OR tattooed[tiab] OR “permanent make-up”[tiab] OR “permanent makeup”[tiab]) #2 ((ink*[tiab] OR pigment*[tiab]) AND (composition[tiab] OR ingredient*[tiab] OR impurity*[tiab] OR “heavy metal*”[tiab] OR “aromatic amine*”[tiab] OR “polycyclic aromatic”[tiab] OR label*[tiab] OR labelling[tiab])) #3 (nanoparticle*[tiab] OR “particle size”[tiab] OR nanomaterial*[tiab] OR aggregat*[tiab] OR agglomerat*[tiab] OR “surface area”[tiab]) #4 (macrophage*[tiab] OR fibroblast*[tiab] OR “mast cell*”[tiab] OR keratinocyte*[tiab] OR dendritic[tiab] OR intracellular[tiab] OR epidermis[tiab] OR dermis[tiab]) #5 (photodegradation[tiab] OR photostability[tiab] OR photodecomposition[tiab] OR sunlight[tiab] OR ultraviolet[tiab] OR UV[tiab] OR metabolism[tiab] OR ageing[tiab] OR aging[tiab]) #6 #1 AND (#2 OR #3 OR #4 OR #5)	639
Embase (Embase.com)	#1 (tattoo OR tattoos OR tattooing OR tattooed OR ‘permanent make-up’ OR ‘permanent makeup’):ti,ab #2 (ink* OR pigment*):ti,ab AND (composition OR ingredient* OR impurity* OR ‘heavy metal*’ OR ‘aromatic amine*’ OR ‘polycyclic aromatic’ OR label* OR labelling):ti,ab #3 (nanoparticle* OR ‘particle size’ OR nanomaterial* OR aggregat* OR agglomerat* OR ‘surface area’):ti,ab #4 (macrophage* OR fibroblast* OR ‘mast cell*’ OR keratinocyte* OR dendritic OR intracellular OR epidermis OR dermis):ti,ab #5 (photodegradation OR photostability OR photodecomposition OR sunlight OR ultraviolet OR UV OR metabolism OR ageing OR aging):ti,ab #6 #1 AND (#2 OR #3 OR #4 OR #5)	Not available
Scopus	TITLE-ABS(tattoo OR tattoos OR tattooing OR tattooed OR “permanent make-up” OR “permanent makeup”) AND (TITLE-ABS((ink* OR pigment*) AND (composition OR ingredient* OR impurity* OR “heavy metal*” OR “aromatic amine*” OR “polycyclic aromatic” OR label* OR labelling)) OR TITLE-ABS(nanoparticle* OR “particle size” OR nanomaterial* OR aggregat* OR agglomerat* OR “surface area”) OR TITLE-ABS(macrophage* OR fibroblast* OR “mast cell*” OR keratinocyte* OR dendritic OR intracellular OR epidermis OR dermis) OR TITLE-ABS(photodegradation OR photostability OR photodecomposition OR sunlight OR ultraviolet OR UV OR metabolism OR ageing OR aging))	Not available
Web of Science Core Collection	#1 TI = (tattoo OR tattoos OR tattooing OR tattooed OR “permanent make-up” OR “permanent makeup”) OR AB = (tattoo OR tattoos OR tattooing OR tattooed OR “permanent make-up” OR “permanent makeup”) #2 (TI = (ink* OR pigment*) OR AB = (ink* OR pigment*)) AND (TI = (composition OR ingredient* OR impurity* OR “heavy metal*” OR “aromatic amine*” OR “polycyclic aromatic” OR label* OR labelling) OR AB = (composition OR ingredient* OR impurity* OR “heavy metal*” OR “aromatic amine*” OR “polycyclic aromatic” OR label* OR labelling)) #3 TI = (nanoparticle* OR “particle size” OR nanomaterial* OR aggregat* OR agglomerat* OR “surface area”) OR AB = (nanoparticle* OR “particle size” OR nanomaterial* OR aggregat* OR agglomerat* OR “surface area”) #4 TI = (macrophage* OR fibroblast* OR “mast cell*” OR keratinocyte* OR dendritic OR intracellular OR epidermis OR dermis) OR AB = (macrophage* OR fibroblast* OR “mast cell*” OR keratinocyte* OR dendritic OR intracellular OR epidermis OR dermis) #5 TI = (photodegradation OR photostability OR photodecomposition OR sunlight OR ultraviolet OR UV OR metabolism OR ageing OR aging) OR AB = (photodegradation OR photostability OR photodecomposition OR sunlight OR ultraviolet OR UV OR metabolism OR ageing OR aging) #6 #1 AND (#2 OR #3 OR #4 OR #5)	Not available

Field codes restrict all searches to titles and abstracts; the asterisk denotes truncation. The symbol # followed by a number denotes a numbered search statement: #1 corresponds to the population core, #2–#5 to thematic blocks A–D (Table 2) and #6 combines them by reference to these numbers. Re-run on 10 September 2026 with the entry date limited to 14 August 2026, the PubMed strings reproduced the yields reported in Section 2 to within two records. The Embase, Scopus and Web of Science strings express the strategy described in Section 2 in the syntax of each platform; their yields were not available for this revision.

**Table A2:** Publications and documents included in the core synthesis (*n* = 33): evidence category, material and principal constraint on inference.

Ref.	Category	Material and Sample	Principal Constraint on Inference
[5]	e	Commission Regulation (EU) 2020/2081: entry 75 and Appendix 13 of Annex XVII to REACH	Limits apply to products placed on the market; no data on deposits in skin
[7]	c	41 commercial inks sold in the EU after 2022; 12 elements	Exposure and cancer risk modelled, not measured in tissue
[8]	c	54 inks of 9 brands, US market	Product analysis limited to the US market
[9]	c	10 blue and green inks from 5 manufacturers, declared REACH-compliant	Small sample; PB 15 polymorph undetermined in 4 inks
[10]	e	52 inks of 26 brands from 14 countries; European enforcement action	Enforcement report, not peer-reviewed
[11]	a	Skin and regional lymph nodes of 4 tattooed and 2 non-tattooed donors, post mortem (synchrotron XRF, FTIR)	Four tattooed donors; laser treatment history not reported
[12]	b	Mouse model with diphtheria toxin-mediated macrophage ablation	Transfer to human skin not demonstrated
[14]	a	Tattoos of 11 subjects imaged in vivo by two-photon FLIM (1 freshly applied, 10 applied more than 3 years earlier)	Carbon black only; small number of subjects
[15]	e	Council of Europe Resolution ResAP(2008)1, including maximum allowed concentrations of impurities	Non-binding recommendations
[45]	c	Tattoo inks in general usage (laser diffraction, electron microscopy, XRD)	Stock inks; no tissue data
[24]	d	JRC Science for Policy report on the safety of tattoos and PMU	Secondary synthesis
[46]	a, c	Tattooed forearm skin of one donor (AFM, light microscopy); human dermal fibroblasts exposed to diluted ink	Single tissue donor
[47]	d	Systematic review of ink ingredients, fate in skin, PAHs, primary aromatic amines, metals and regulation	Secondary evidence
[48]	c	Py-GC/MS library of 36 organic pigments; 28 commercial inks and 18 laboratory mixtures	Method validation; no tissue data
[18]	b	Porcine model (3 tattooed animals, 1 control), 28 days; ICP-MS of skin, lymph nodes and organs	Elements only; three animals
[19]	c	Two azo pigments in suspension before and after laser irradiation (HPLC, MS)	Suspension experiment; no tissue data
[20]	a	Excised porcine and human skin tattooed with PR 22 under various conditions	Ex vivo tattooing; values immediately after tattooing only
[21]	a	Red pigments extracted from 9 tattooed human skin specimens and quantified in 5 (HPLC)	Loss estimated by comparison with [28], not measured longitudinally
[49]	c	86 ink samples; LC-QqQ-MS screening for 40 substances of high concern	Product analysis; no tissue data
[50]	d	Survey of European studies on aromatic amines in about 300 ink samples, including an Italian surveillance programme	Secondary summary
[51]	c	19 commercial tattoo inks (HPLC, MS); keratinocytes exposed to ink extracts and UVA	Product analysis and cell culture; no tissue data
[22]	c	Recovery of PR 22, PR 9 and their decomposition products from suspension and lysed skin (HPLC-DAD)	Method development
[30]	b	SKH-1 mice tattooed with PR 22; laser and simulated solar exposure	Mouse model
[52]	a	Human biopsies taken from 24 h to 40 years after tattooing (electron microscopy)	Descriptive ultrastructural study
[53]	b	Mice injected with India ink particles and latex beads (light and electron microscopy)	Model particles rather than commercial tattoo inks
[26]	b	Tail skin of tattooed mice, including macrophage-depleted animals	Mouse model
[28]	a	Untreated and Q-switched ruby laser-treated human tattoos (light and electron microscopy)	Localisation in untreated tattoos described qualitatively
[27]	a	19 biopsies of red tattoo reactions (histopathology, immunohistochemistry)	Reaction-selected specimens
[54]	c	Two-step Py-GC/MS protocol for ink ingredients and counterfeit products	Methods protocol
[23]	c	41 coloured tattoo pigments (structural and spectroscopic analysis)	Product analysis; no tissue data
[55]	d	Summary of the photostability and breakdown products of pigments used in tattoo inks	Largely in vitro data
[56]	c	7 yellow tattoo inks; photodecomposition of PY 74 in tetrahydrofuran under simulated solar light	Solution chemistry; no tissue data
[57]	d	Systematic review of degradation products: 11 photolysis and 2 metabolism studies	Few primary studies

Evidence categories: (a) human tissue in vivo, in biopsies, post mortem or in excised skin; (b) animal model; (c) inks, pigments or extracts analysed outside tissue, including suspensions, solutions and cell cultures; (d) secondary source; (e) normative or enforcement document. AFM, atomic force microscopy; FLIM, fluorescence lifetime imaging; FTIR, Fourier-transform infrared spectroscopy; ICP-MS, inductively coupled plasma mass spectrometry; JRC, Joint Research Centre; MS, mass spectrometry; PR, Pigment Red; Py-GC/MS, pyrolysis–gas chromatography/mass spectrometry; UVA, ultraviolet A; XRF, X-ray fluorescence.

**Table A3:** Status under entry 75 of Annex XVII to the REACH Regulation of the pigments named in the cited studies.

Pigment (C.I. Number)	Category	Studies
Pigment Red 9 (12460)	1	[19,22]
Pigment Red 22 (12315)	1	[19,20,21,22,30]
Pigment Red 112 (12370)	1	[21]
Pigment Orange 13 (21110)	1	[4,11]
Pigment Yellow 14 (21095)	1	[32]
Pigment Yellow 65 (11740)	1	[32]
Pigment Yellow 74 (11741)	1	[32,56]
Pigment Blue 15:3 (74160)	2	[3,9,55]
Pigment Green 7 (74260)	2	[9,11,55]
Pigment Red 170	3	[11]
Quinacridone pigment (73915)	3	[55]
Dioxazine pigment (51319)	3	[55]
Carbon black (Pigment Black 7)	3	[14,51]
Titanium dioxide (Pigment White 6; 77891)	3	[11,29,32]

Category 1, listed individually in Appendix 13 to Annex XVII–Annex XVII to the REACH Regulation [5] (concentration limit 0.1% by weight, applicable after 4 January 2022); category 2, subject to paragraph 1 of entry 75 only after the derogation in paragraph 4 expired on 4 January 2023; category 3, not named in entry 75 or Appendix 13, so that any restriction depends on the generic hazard-based criteria of paragraph 1 of entry 75, which were not assessed for individual pigments in this review [5]. C.I., Colour Index.
